# A three-level meta-analysis of the association between students’ epistemic cognition and learning outcomes in South Korea

**DOI:** 10.3389/fpsyg.2026.1841294

**Published:** 2026-08-04

**Authors:** Jihyun Song, Wonseok Choi

**Affiliations:** Department of Kinesiology, Keimyung University, Daegu, Republic of Korea

**Keywords:** academic achievements, epistemic beliefs, epistemic cognition, epistemological beliefs, epistemology, student learning

## Abstract

**Introduction:**

In the current era where unverified and non-credible information is prevalent, students’ epistemic cognition has become increasingly critical in education. Epistemic cognition helps students develop the abilities to acquire, understand, justify, change, and use knowledge. Although the literature has provided extensive insights into epistemic cognition, the association between such cognition and learning outcomes remains unclear within specific cultural contexts. Given that epistemic cognition is particularly shaped by the culture, the purpose of this study was to synthesize empirical evidence on the association between students’ epistemic cognition and learning outcomes in South Korea through a three-level meta-analysis.

**Methods:**

Following the PRISMA guideline, this study conducted a three-level meta-analysis. A hierarchical model was employed to account for statistical dependence across 512 effect sizes from 18 peer-reviewed studies. Multivariate meta-regression and sensitivity analyses were performed to determine significant moderators and ensure the robustness of the pooled effect sizes.

**Results:**

The analysis yielded a statistically significant small-to-moderate pooled effect size (*r* = 0.191, *p* < 0.001). The heterogeneity test exhibited low-to-moderate variance at Level 2 (*τ^2^* = 0.015, *I^2^* = 35.82%) and moderate variance at Level 3 (*τ^2^* = 0.024, *I^2^* = 56.64%). The school level and epistemic cognition construct were found to be key moderators of the pooled effect size, while the other four, domain specificity, alignment, scale polarity, and type of learning outcome, were not.

**Conclusion:**

These findings suggest that epistemic cognition is a critical factor in relation to different types of learning outcomes, such as higher and lower order cognitive outcomes, self-regulation, and motivation. The school level and epistemic cognition constructs appear to influence the association between epistemic cognition and learning outcomes. However, caution should be taken in interpreting these findings, given that the effect sizes were based on correlation coefficients from Korean studies, limiting causal inference and generalizability across different contexts and cultures.

## 1 Introduction

The development of digital technologies enables the global population to have access to large amounts of data and information quickly and easily. In particular, generative artificial intelligence (GenAI), such as ChatGPT, Claude, and Google Gemini, has served as a powerful means for learning, teaching, and curriculum design in education (OECD, 2022). A critical issue, however, is that the information generated by GenAI is neither verified nor credible, often producing fake, misleading, or inaccurate content (Eysenbach, 2023). In today’s world, “greater information and knowledge come with greater uncertainty,” which makes the capabilities to evaluate and justify knowledge central to learners (OECD, 2022, p. 9). Such capabilities require learners to develop epistemic cognition, defined as a cognitive process that individuals “acquire, understand, justify, change, and use knowledge” (Greene et al., 2016, p. 1).

Epistemic cognition has been studied in the fields of education and psychology for over 55 years; the development of digital technologies has accelerated attention to the topic. Epistemic cognition has primarily been conceptualized as developmental and multidimensional (Hofer and Pintrich, 1997). Initial researchers (e.g., Kuhn, 1991; Perry, 1970) assumed that epistemic cognition develops with increases in age and educational levels. For example, Perry (1970) as a pioneer of a developmental model proposed the developmental stage of epistemic cognition, ranging from *dualism* (dichotomous/absolute view of knowledge as either truth or not) to *commitment within relativism* (views of knowledge and knowing as shaped by the values and identities individuals support) through *multiplicity* (modified dichotomous view of knowledge by acknowledging uncertainty), and *relativism* (view of knowledge and knowing as relative, context-dependent). These stages have been further expanded from a multidimensional perspective, which posits that epistemic cognition comprises relatively independent belief dimensions/constructs. Schommer (1990) suggested five independent constructs of epistemic cognition, including beliefs about (a) *certainty of knowledge*, ranging from certain/absolute to uncertain/tentative, (b) *structure of knowledge*, ranging from simple/isolated to integrated, (c) *source of knowledge*, ranging from handed down by authority (e.g., teachers, experts) to obtained from observation and reason, (d) *speed of learning*, ranging from quick-all-or-non to gradual, and (e) *ability to learn*, ranging from entity/fixed at birth to incremental/lifelong-improving. Based on the five epistemic constructs, Jehng et al. (1993) broadened the simple beliefs to those about *rigid learning*, ranging from a process in which individuals passively acquire already-formulated, simple knowledge to one in which they develop integrated knowledge constructs on their own. Reviewing previous studies on developmental and multidimensional epistemic beliefs, Hofer and Pintrich (1997) reconceptualized the epistemic cognition constructs as the nature of knowledge (certainty, simplicity) and the nature of knowing (source, justification), while proposing the nature of learning (learning speed, ability to learn) as peripheral to core epistemic cognition. Beliefs about *justification for knowing* present a way in which individuals evaluate knowledge claims, such as the use of evidence, authority, or expertise. From the multidimensional perspective, the more individuals perceive knowledge and knowing as uncertain/tentative, integrated, evidence-, observation-, and reason-based, gradual, and incremental/lifelong-improving, the more they possess “sophisticated” epistemic cognition. In contrast, viewing knowledge and knowing as certain, simple, and dictated by authority, quick-all-or-non, and entity/fixed reflects “naïve” epistemic cognition (Schommer, 1990). Since the multidimensional aspect of epistemic cognition was proposed, the conceptualization of epistemic beliefs has been further expanded to encompass diverse constructs, such as epistemic aims, epistemic values, and epistemic ideals (Chinn et al., 2011).

Scholars have debated whether epistemic cognition is general or specific (Hofer and Pintrich, 1997; Muis et al., 2006). *Domain generality* posits that epistemic cognition is consistent/general across academic domains. *Domain specificity* assumes that epistemic cognition varies in an academic domain from others, such as science, mathematics, and history. Although the debate is ongoing, previous studies have suggested that epistemic cognition is both general and specific, with similarities and differences across academic domains (Muis et al., 2006; Rosman et al., 2020). For example, synthesizing 19 empirical studies, Muis et al. (2006) found evidence of domain generality, based on positive correlations in epistemic beliefs across academic domains such as social studies, mathematics, and business. The study also revealed domain specificity by identifying significant mean differences in epistemic beliefs between science (e.g., physics, chemistry) and psychology. The study further suggested that epistemic cognition is even topic/context specific within an academic domain, providing a more nuanced understanding of epistemic cognition.

The importance of understanding students’ epistemic cognition is its ability to explain learning outcomes in education. Previous studies have confirmed a positive association between epistemic cognition and learning outcomes, such as academic achievement (Greene et al., 2018), self-efficacy (Muis and Duffy, 2013), and motivation (Chen, 2012). In a meta-analysis, for example, Greene et al. (2018) found that students’ epistemic cognition across countries was associated with learning outcomes, including argumentation, conceptual change, and declarative and procedural knowledge, with a small-to-moderate effect size. Chen (2012) reported that epistemic cognition of 1,225 middle and high school students in the U. S. was associated with motivational constructs, such as self-efficacy and mastery goal orientations. These findings suggested that the more sophisticated epistemic beliefs are positively associated with enhanced learning outcomes.

The association between epistemic cognition and learning outcomes differs across learning contexts (Muis et al., 2016). Learning contexts are often created by teachers’ instructional approaches, where the cognitive and psychological dynamics of epistemic cognition are activated. Bendixen and Rule (2004) suggested that learning contexts should include various activities that help learners become aware of cognitive disequilibrium (doubt about their existing epistemic cognition), perceive epistemic volition (a desire to resolve that doubt), and employ resolution strategies (reflection, discussion, and argumentation). Such contexts are often embedded in constructivist learning approaches in that they are central to students’ active engagement in learning to construct their knowledge from individual and interpersonal experiences (Vygotsky, 1978). Previous studies have confirmed that constructivist instructional contexts, such as inquiry-based instruction, source evaluation, and metacognitive scaffolds, help students develop sophisticated epistemic beliefs and learning achievement (Cartiff et al., 2021; Muis and Duffy, 2013).

Although the research evidence on the association between epistemic cognition and learning outcomes is well-documented across countries/cultures, a majority of studies were conducted in Western countries, leaving a lack of a nuanced understanding of the cultural-specific association (see Greene et al., 2018 for details). Scholars have argued that epistemic cognition and its relevance to learning outcomes cannot be translated from Western to other countries without considering their cultural complexity as epistemic cognition is socially and culturally constructed (Bang and Medin, 2010; Hofer and Pintrich, 1997). One of the key cultural distinctions in the literature is the individualism–collectivism framework (Hofstede, 1991). In individualistic cultures, it is assumed that individuals are autonomous, independent subjects with unique perspectives, conceptualizing learning as an active, student-centered process of inquiry (Felbrich et al., 2012; Youn et al., 2001). Learners within the cultures are expected to navigate epistemic uncertainty, engage in critical discussion, and question authority, which, in turn, fosters higher-order and critical thinking. In contrast, collectivistic cultures often prioritize individuals as socially interdependent to conform to collective goals, shared relational norms, and authority (Felbrich et al., 2012; Youn et al., 2001). Within the cultures, teacher-centered education is emphasized, and learners passively receive absolute knowledge, adhere to relatively strict rules, and comply with instructional authority. However, this individualism–collectivism framework does not consistently account for learners’ epistemic cognition and its association with learning outcomes. For example, Felbrich et al. (2012) demonstrated that learners in Taipei and Singapore considered collectivistic cultures possessed both sophisticated and naïve epistemic beliefs. Similar findings were observed in cross-cultural comparison studies (e.g., Youn et al., 2001), suggesting that in certain Asian cultures learners’ epistemic cognition is highly nuanced and complex, which cannot be captured by the single cultural label. In addition, Asian societies such as Singapore, Taipei, Japan, and South Korea, traditionally classified as collectivistic cultures, have consistently achieved top-tier rankings in the Programme for International Student Assessment (PISA) as an international standardized test (OECD, 2023), suggesting that the psychological and cognitive mechanisms of academic success in Asian societies are more dynamic and complex. Moving beyond macro-cultural generalizations, it is thus necessary to understand epistemic cognition and its association with learning outcomes within a very specific culture (Park and Han, 2018).

Among Asian countries, South Korea (Korea hereafter) in particular likely offers a unique cultural milieu for investigating such epistemic dynamics and their relevance to learning outcomes. Historically, within the interdependent social structure, the high authority of teachers has played a central role in shaping students’ epistemic cognition (Youn, 2000). This cultural aspect is well encapsulated in the Korean saying, “One should not even step on the shadow of a teacher.” This suggests that teachers serve as role models for behavior and knowledge to be followed (Choi and Kwon, 2012). Within the context, students tend to cultivate absolute knowledge through conformity to authority figures, shaping their beliefs about knowledge and knowing. However, this cultural environment has rapidly changed due to industrialization and Westernization over recent decades, creating a coexistence of individualism and collectivism (Park and Han, 2018; Youn et al., 2001). Specifically, within the culture, while the older generation tends to conform to in-group norms, the younger generation tends to pursue subjective selfhood, individuality, and other-focused, empathetic relationship formation (Park and Han, 2018). In addition, the Ministry of Education (2022) has consistently mandated in the national curriculum a systematic transition from teacher-centered, one-way lecturing and students’ passive, repetitive, and rote learning to learner-centered, constructivist learning by facilitating inquiry-based learning, critical thinking, conceptual understanding, and problem-solving to strengthen national competitiveness (Choi, 2016). This top-down policy, however, appears constrained in actual practice; as students progress to higher grade levels, they need to prepare for the Sooneung (the high-stakes national college entrance examination), which rigorously reinforces an exam- and assessment-oriented learning climate (Park and Kim, 2021; Youn et al., 2001).

Given these unique cultural and educational dynamics in Korea, where older generation’s high respect for and conformity to authority, younger generation’s pursuit of individuality and freedom, modern constructivist instructional mandates in the national curriculum, and intense examination-focused competition coexist, students’ epistemic cognition in the unique learning context may vary in its function and relevance to learning outcomes, suggesting the need to synthesize the existing body of research in order to clarify how epistemic cognition operates in this distinctive context. To our knowledge, although scholars have attempted to synthesize a large volume of the literature, a majority of the reviews focused on conceptual definitions, theoretical frameworks, and expansions of the epistemic belief constructs (Greene et al., 2016; Hofer, 2016; Hofer and Pintrich, 1997; Kelly, 2021). Consequently, robust empirical evidence synthesizing the association between epistemic cognition and learning outcomes remains surprisingly sparse. Recently, Greene et al. (2018) provided empirical evidence for the association between epistemic cognition and learning outcomes through a meta-analysis; but the macro-level synthesis based on data drawn from diverse cultural contexts offered limited insight into how the association is observed within specific non-Western learning contexts, such as Korea. Given Korea’s distinctive cultural and educational dynamics described above, it is necessary to clarify the nature and strength of these associations within the Korean learning context. Therefore, the purpose of this study was to synthesize empirical evidence on the association between students’ epistemic cognition and learning outcomes in the Korean learning context. This study adopted a three-level meta-analysis approach to address the dependent issue in effect sizes and include all effect sizes reported within a study (Cheung, 2014; Van Den Noortgate et al., 2013). The findings of this study would provide critical insights into whether the association between students’ epistemic cognition and learning outcomes within the Korean learning context. In doing so, the study may contribute to ongoing theoretical discussions regarding the cultural generalizability and contextual dependency of epistemic cognition. In addition, the findings may inform educational policy and instructional, curriculum, and intervention designs aimed at fostering students’ epistemic cognition in the culturally dynamic learning contexts beyond Korea with similar cultural and educational characteristics.

## 2 Methods

This study followed the Preferred Reporting Items for Systematic Reviews and Meta-Analyses (PRISMA) 2020 statement. PRISMA is a reporting guideline, which comprises a 27-item checklist designed to ensure transparent, rigorous, and comprehensive reporting for systematic and meta-analytic reviews (Page et al., 2021). Following this guideline, this study established inclusion criteria and searched multiple electronic databases. Then, data were extracted from the included studies, their quality was assessed, and analyses were conducted to demonstrate the relationship between students’ epistemic cognition and learning outcomes.

### 2.1 Inclusion criteria

This review included studies that met the following criteria: (a) written in English or Korean; (b) published in peer-reviewed journals before November 2025; (c) a sample of K-12 students, ranging from 1st to 12th graders, in the Korean school context; (d) measuring the association between epistemic cognition (e.g., beliefs about certainty, simplicity, source, justification, learning speed, ability to learn) and learning outcomes (e.g., standardized test scores, critical thinking, conceptual understanding, self-regulation, learning strategies, motivation); and (e) necessary statistical data reported, including correlation coefficients (*r*s) and standardized regression analysis coefficients (*β*s) with sample size. As this study aimed to synthesize the association between students’ epistemic cognition and learning outcomes, both experimental and non-experimental studies were eligible for this meta-analysis if they met the criteria. Studies that did not meet these criteria were excluded, such as those involving university students or pre- and in-service teachers; those conducted outside the Korean school context; those that did not report the necessary statistics; and gray literature (e.g., theses, dissertations, or conference papers).

### 2.2 Literature search strategy

Nine electronic databases were used to conduct a structured, systematic literature search: Korea Citation Index (KCI), Korean Studies Information Service System (KISS), Web of Science, Scholar, DBpia, eArticle, Education Source, Scopus, and ERIC. Initial search strings to identify potential studies included the following keywords: (“epistemic” OR “epistemological”) AND (“cognition” OR “beliefs”) AND (“students” OR “elementary school” OR “middle school” OR “high school”) AND (“learning outcomes” OR “learning achievement” OR “academic achievement” OR “cognitive” OR “affect” OR “motivation” OR “behavior” OR “engagement”) at an abstract level. However, this initial search yielded 10 studies, likely due to terminological heterogeneity in how epistemic cognition and learning outcomes were described in the original studies. In addition, given that this study was the first meta-analysis to synthesize Korean students’ epistemic cognition, expanding the search strategy without restricting the start date was necessary to improve search sensitivity and avoid omitting relevant studies. Thus, focusing on the topic of epistemic cognition, we decided to use the following search strings: (“personal epistemology”) OR (“epistemic” OR “epistemological”) AND (“cognition” OR “beliefs” OR “views”) at the abstract level in the Korean databases. When searching the literature in the international databases (e.g., Education Source, Scopus, ERIC), “Korea” was added to the search strings to ensure the scope of the study. This updated search was conducted up to February 10, 2026.

### 2.3 Data extraction and collection

Before extracting data from the included studies, we independently reviewed five studies and developed a coding sheet. We then shared the coding sheets and discussed discrepancies to ensure inter-rater reliability. For example, we found that epistemic cognition constructs were measured using either a bipolar scale (e.g., certainty measured on a single continuum from certain to fluid beliefs) or a separate scale (measuring each side of the beliefs independently). Accordingly, we created a coding category (i.e., scale polarity) with two codes (i.e., bipolar, separate). In addition, we classified learning outcomes into four categories, including higher order cognitive outcome, lower order cognitive outcome, motivation/affect, and self-regulation based on Bloom’s (1956) classical taxonomy. In particular, although self-regulation is part of higher-order thinking, we distinguished it from the category, higher-order cognitive outcome, because self-regulation functions as activating both cognitions and behaviors through which learners monitor and control their learning processes, whereas higher-order cognitive outcomes were treated as the educational products that learners ultimately proved in measures (Dinsmore et al., 2008; Schunk, 2008). Using the coding sheet, we extracted data from the complete datasets of the studies (*n* = 18). We also collected statistics (e.g., *r, β*) and sample sizes during the data collection process. In a subsequent meeting, we discussed further discrepancies. For instance, Yoo and Yum (2019) treated perceived utility of mathematics, problem-solving, and self-efficacy as components of epistemic cognition; these factors are theoretically recognized as learning outcomes influenced by epistemic cognition in a learning context (Hofer and Pintrich, 1997; Greene et al., 2018). Therefore, we decided to assign these factors to the learning outcome category for conceptual and theoretical alignment. The finalized coding sheet, including 13 code categories and 73 codes, is described in Table 1.

**Table 1:** Coding sheet for data collection.

Code categories	Specific codes
School level	Elementary school, middle school, high school, mixed (middle and high school)
Grade	4th–6th, 6th, 7th, 7th–9th, 7th–12th, 8th, 10th, 11th, 12th
Gender	Mixed (both female and male), only female, not reported
Epistemic cognition construct	Certainty, simplicity, source, justification, ability to learn, learning speed, learning process, beliefs about knowledge, beliefs about learning
Learning outcome	
Higher order cognitive outcome	Cognitive conflict, conceptual understanding, critical thinking, information literacy, mathematical problem solving, acceptance of evolution theory, multiple document selection, multiple document use, scientific inquiry skill
Lower order cognitive outcome	Standardized tests (overall, Korean language, mathematics, science, social studies), evolution content knowledge
Motivation/affect	Matery-approach goal, mastery-avoidance goal, performance-approach goal, performance-avoidance goal, implicit theory of intelligence, intercultural sensitivity, situational interest, learning motivation, perceived utility of mathematics, mathematical self-efficacy, academic stress, test anxiety, group activity preference, scientific attitude
Self-regulation	Meta-cognition, self-regulated learning, deep learning strategy, surface learning strategy, learning style, mathematics test preparation, academic emotion regulation, frequency of asking questions
Domain specificity	Domain-general, domain-specific, content-specific
Academic subject	Mathematics, science, social studies
Scale polarity	Bipolar scale, separate scale
Alignment	Aligned, non-aligned

### 2.4 Quality assessment

To assess the quality of the included studies, we used the Quality Assessment Tool for Observational Cohort and Cross-Sectional Studies (National Institutes of Health, 2020). The tool consists of 14 items designed to evaluate the internal validity of both types of the studies. In the present study, however, Items 6, 7, 10, 12, and 13 were excluded from this assessment, as they are intended to assess the quality of longitudinal or cohort studies that measure variables at multiple time points. We coded each remaining item as “Yes” if the study met the criterion, “No” if not, and “NR” if not reported. After assessing each item, we aggregated the scores to determine the final quality rating. Studies were rated as “Good,” “Fair,” and “Poor” if their aggregated “Yes” scores were 7–9, 4–6, and 0–3, respectively.

### 2.5 Data analysis

Traditional meta-analyses have relied on univariate approaches that treat multiple effect sizes within a study as independent, select one effect size, or average them into a single estimate per study. However, these approaches do not account for the statistical dependence inherent in data in which multiple effect sizes are extracted from the same sample (Cheung, 2014). Given that all studies included in the current analysis reported more than three effect sizes, a more robust approach was required. Thus, we adopted a three-level meta-analysis approach. This approach partitions the total variance into three levels: Level 1 explains the sampling error of the observed effect sizes; Level 2 accounts for within-study variance (differences of effect sizes within a study); and Level 3 represents between-study variance (differences of effect sizes across independent studies) (Cheung, 2014; Van Den Noortgate et al., 2013). In this study, Level 1 variance would be determined by the sample size within each study. Level 2 variance would arise from differences in the measurement of epistemic cognition constructs and learning outcomes across multiple effect sizes within a study. Level 3 variance would reflect differences in study characteristics, such as school levels, grades, gender, domain specificity, and measurement instruments. Adopting this hierarchical structure enables all relevant effect sizes to be used in the meta-analysis without violating the independence assumption.

Before data analysis, we transformed *β*s to *r*s to ensure that all data were consistent across studies. It has been acknowledged that *β*s can be converted if they range from −0.50 to 0.50. In the dataset, this transformation was applied to a single study (Choi, 2019), which reported only 24 *β*s, ranging from −0.20 to 0.23. We calculated *r*s from these *β*s using the Peterson and Brown’s (2005) formula: *r* = *β* + 0.05*λ*, where λ is 1 if *β* ≥ 0, or λ is 0 if *β* < 0. We then converted all *r*s into Fisher’s *z*-scores because *r*s are heavily dependent on their variances, limiting their direct synthesis (Borenstein et al., 2009). After conducting all analyses, we transformed Fisher’s *z*-scores back to *r*s to report the results. The *r*s were interpreted as weak if > 0.1, moderate if > 0.3, and strong if > 0.5 (Cohen, 1988). For studies reporting effect sizes in the opposite direction due to the nature of the measurements, the directions were inverted. Specifically, for effect sizes involving naïve epistemic cognition constructs (e.g., beliefs about certainty, simplicity, source, justification) and/or negative learning outcomes (e.g., surface learning strategies, academic stress, or test anxiety), the signs were reversed by multiplying by −1. This alignment ensures consistency across all synthesized effect sizes, indicating the consistent association between epistemic cognition and learning outcomes.

The three-level meta-analysis was conducted in R (v. 4.5.0) using the rma.mv function of the metafor package. The pooled estimate of effect sizes was computed using a restricted maximum likelihood (REML) estimator. Heterogeneity of the pooled estimate was assessed using the *Q*-statistic and *I^2^* statistics. The magnitude of variance across the three levels was calculated using *I^2^* for the proportion of variance and $\tau^{2}$ for the absolute amount of true heterogeneity for each level. A Log-Likelihood Ratio Test (LRT) was also performed to justify using a three-level meta-analysis rather than a traditional two-level meta-analysis. These statistics were interpreted as follows: potential heterogeneity if the *Q*-statistic was significant (*p* < 0.05); *I^2^* values of 25, 50, and 75% as low, moderate, and high, respectively; and a significant LRT result (*χ^2^*, *p* < 0.05) indicating both significant heterogeneity ( $\tau^{2}$ > 0) and a better fit for the three-level meta-analysis than the two-level meta-analysis (Cheung, 2014; Higgins et al., 2003).

The three-level model was extended to a three-level mixed-effects model to demonstrate the contribution of pre-identified categorical moderators during data collection to the association between epistemic cognition and learning outcomes, using multivariate meta-regression (Assink and Wibbelink, 2016). This model treats moderators as fixed-effects, while effect sizes are considered to differ randomly across and within studies, allowing for explaining residual heterogeneity that remains after the fixed-effects are included (Borenstein et al., 2009). The *F*-test was reported to determine the significant contribution of moderators. Subgroup analyses were also performed to estimate the specific effect sizes within each moderator. Potential publication bias was assessed through a funnel plot and a three-level extension of Egger’s regression test (Fernández-Castilla et al., 2021). Potential publication bias was expected to be minimal or undetected if the funnel plot exhibited a symmetric distribution with a statistically insignificant slope of the Egger’s test (*p* > 0.05), where the standard error was incorporated as a moderator within the 3-level model. In addition, the intercept was examined to determine whether the model-implied effect size, as calculated when the standard effort was 0, remained robust after controlling for potential publication bias. Two types of sensitivity analyses were conducted: one using a leave-one-out method to assess whether any single study influenced the pooled estimate, and another using standardized residuals to examine extreme effect sizes (outliers) exceeding ±z = 3.29.

## 3 Results

### 3.1 Study selection process

**Figure 1:**
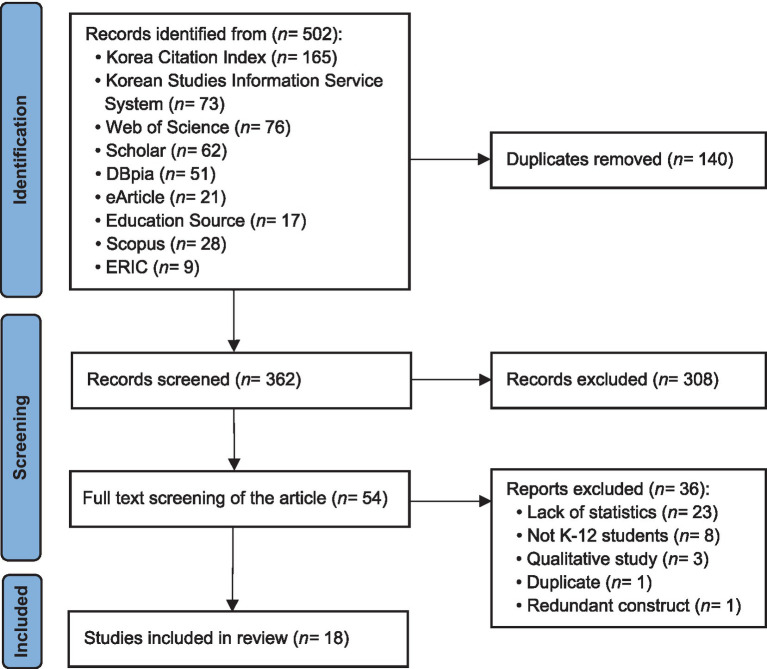
Flow diagram of literature search process.

Figure 1 exhibits a flow diagram of the study selection process. From the nine databases, we initially identified 502 studies. After removing duplicates (*n* = 140), we screened 362 potential studies, of which 308 were excluded based on the titles and abstracts. Then, we reviewed 54 full-text studies based on the inclusion criteria, of which 36 were excluded due to lack of statistics (*n* = 23), not being K-12 students (*n* = 8), qualitative study (*n* = 3), duplicate (*n* = 1), and redundant measure construct (*n* = 1). In turn, we yielded a total of 18 studies for the meta-analysis.

### 3.2 Characteristics of the included studies

Table 2 exhibits the characteristics of the 18 included studies. The total sample size across the studies was 6,312, ranging from 87 to 1,086 (*M* = 350.67, *Mdn* = 304.00). Most studies (*n* = 17) adopted a non-experimental research design, while one study used a quasi-experimental design (Kang et al., 2007). Two studies reported sample sizes by specific groups, separately, such as academic tracks (*n* = 166 for the Humanities track and *n* = 174 for the Science track; Yoon et al., 2014) and religious affiliation (*n* = 46 for Christians and *n* = 139 for non-Christians; Kim, 2015). Most studies (*n* = 14) included mixed-gender participants, while one involved only females, and three did not report gender distribution. School levels were identified as elementary school (*n* = 4), middle school (*n* = 5), high school (*n* = 8), and a combination of middle and high schools (*n* = 1). One study (So, 2013) reported effect sizes separately by sample and grade level. Most studies (*n* = 13) adopted domain-general measures of students’ epistemic cognition, while five focused on domain-specific epistemic cognition in science (*n* = 3), mathematics (*n* = 1), and social studies (*n* = 1). One study (Kim, 2015) measured both domain-specific and content-specific epistemic cognition. Following multidimensional models (e.g., Hofer and Pintrich, 1997; Schommer, 1990), most studies (*n* = 14) measured different epistemic cognition constructs, such as beliefs about certainty, simplicity, source, justification, ability to learn, and learning speed. Four studies (Bak and Chai, 2006; Kim, 2015; Kim et al., 2025; Mun, 2009) aggregated the scores of the distinct constructs and reported a total epistemic cognition score. Based on the constructs suggested by Jehng et al. (1993), one study (Youn et al., 2001) developed two constructs: beliefs about learning, which integrated ability to learn and learning speed, and beliefs about knowledge, which combined certainty, source, and learning process. These constructs were measured on either a bipolar scale (*n* = 13) or separate scales that distinguished sophisticated and naïve epistemic beliefs (*n* = 5). Most studies (*n* = 14) indicated alignment of domain specificity between epistemic cognition and learning outcomes, suggesting that epistemic beliefs and learning outcomes were consistently matched in being either domain-general or domain-specific. For learning outcomes, motivation/affect were most frequently measured (*n* = 14), followed by higher order cognitive outcome (*n* = 9), self-regulation (*n* = 8), and lower order cognitive outcome (*n* = 6).

**Table 2:** Characteristics of the included studies.

Study ID	Author	School level	Grade	Sample N	Gender	Domain specificity	Subject	EC model	EC construct	Scale polarity	Alignment	Learning outcome
1	Bak and Chai (2006)	High	11th	398	Mixed	D-general		Hofer (2016), Schommer (1990)	Certainty, simplicity, source, justification, ability to learn, learning speed	Separate	Aligned	Motivation/affect
2	Chang (2022)	High	12th	450	Not reported	D-general		Hofer (2016) and Schommer (1990)	Total EC	Bipolar	Non-aligned	Higher order cognitive outcome, lower order cognitive outcome
3	Choi (2019)	Mid & High	7th–12th	287	Mixed	D-general		Hofer (2016) and Schommer (1990)	Certainty, simplicity, source, justification, ability to learn, learning speed	Separate	Aligned	Motivation/affect
4	Choi and Kwon (2012)	Mid	7th–9th	1,086	Mixed	D-general		Chan and Elliot (2002)	Certainty, source, ability to learn, learning speed	Bipolar	Aligned	Self-regulation
5	Kang et al. (2007)	Mid	7th	218	Not reported	D-specific	Science	Schommer et al. (1990)	Certainty, ability to learn, learning speed	Bipolar	Aligned	Higher order cognitive outcome, motivation/affect, self-regulation
6	Kim (2015)	High	11th	46 (Christian), 139 (Non-Christian)	Mixed	D-specific, C-specific	Science	Tsai and Liu (2005)	Total EC	Bipolar	Aligned	Higher order cognitive outcome, lower order cognitive outcome
7	Kim et al. (2025)	Elementary	4th–6th	309	Mixed	D-specific	Science	Hofer and Pintrich (1997), Jehng et al. (1993), Schommer (1990)	Total EC	Bipolar	Non-aligned	Motivation/affect, self-regulation
8	Kim and Chung (2015)	High	12th	450	Mixed	D-general		Jehng et al. (1993)	Certainty, source, ability to learn, learning speed, rigid learning	Bipolar	Non-aligned	Higher order cognitive outcome, lower order cognitive outcome, self-regulation
9	Kim and Yun (2009)	Mid	8th	118	Female	D-general		Hofer (2016), Schommer (1990)	Certainty, simplicity, source, justification, ability to learn, learning speed	Separate	Aligned	Higher order cognitive outcome
10	Mun (2009)	Elementary	6th	280	Mixed	D-general		Hofer (2016), Schommer (1990)	Total EC	Bipolar	Aligned	Lower order cognitive outcome, motivation/affect, self-regulation
11	Mun and Koh (2009)	High	10th	280	Mixed	D-general		Hofer (2016), Schommer (1990)	Certainty, simplicity, source, justification	Separate	Aligned	Lower order cognitive outcome, self-regulation
12	So (2013)	Elementary	4th, 6th	236 (4th graders), 240 (6th graders)	Mixed	D-general		Schommer (1990)	Ability to learn, learning speed	Bipolar	Aligned	Motivation/affect, self-regulation
13	Song and Jeong (2012)	Mid	7th	87	Mixed	D-general		Hofer (2016), Schommer (1990)	Certainty, simplicity, source, justification, ability to learn, learning speed	Separate	Aligned	Higher order cognitive outcome
14	Yoo and Yum (2019)	Elementary	6th	810	Mixed	D-specific	Math	Hofer and Pintrich (1997), Schommer (1990)	Ability to learn, learning speed	Bipolar	Aligned	Higher order cognitive outcome, motivation/affect, self-regulation
15	Yoon et al. (2014)	High	12th	166 (Humanities track), 174 (Science track)	Not reported	D-general		Jehng et al. (1993)	Certainty, source, justification, ability to learn, learning speed, rigid learning	Bipolar	Aligned	Self-regulation
16	Youn et al. (2001)	High	10th–12th	455	Mixed	D-general		Jehng et al. (1993)	Beliefs about learning, beliefs about knowledge	Bipolar	Aligned	Lower order cognitive outcome, motivation/affect
17	Yu (2003)	High	12th	227	Mixed	D-general		Schommer (1990)	Certainty, simplicity, ability to learn, learning speed	Bipolar	Non-aligned	Lower order cognitive outcome
18	Yu (2020)	Mid	8th	434	Mixed	D-specific	Social studies	Hofer (2016), Schommer (1990)	Certainty, simplicity, source, justification, ability to learn, learning speed	Bipolar	Aligned	Lower order cognitive outcome, higher order cognitive outcome

Sample N, sample number; Mid, middle school; D-general, domain-general; D-specific, domain specific; C-specific, content-specific; EC, epistemic cognition.

### 3.3 Quality assessment of the included studies

Table 3 exhibits the results of the quality assessment of the included studies. Of the 18 studies, 11 (61.1%) were rated as good quality, seven (38.9%) as fair, and none as poor. All the studies with good quality met the criteria for: clearly stated research questions/objectives (Item 1), defined study population (Item 2), uniform inclusion and exclusion criteria (Item 4), different levels of independent variables examined (Item 8), valid, reliable, and consistent measurement of independent variables (Item 9), and dependent variables (Item 11). Most of the good-quality studies (*n* = 8, 72.7%) provided participation rates of eligible people (Item 3), and three studies (27.3%) provided sample size justifications (Item 5). Notably, three studies (27.3%) met the criterion for statistical adjustment for confounding variables (Item 14). In contrast, seven fair-quality studies mostly met Items 1, 2, 4, 8, 9, and 11, but commonly lacked information on Items 3, 5, and 14.

**Table 3:** Results of quality assessment of the included studies.

Author (Year)	Q1	Q2	Q3	Q4	Q5	Q8	Q9	Q11	Q14	Rating
Bak and Chai (2006)	Yes	Yes	Not reported	Yes	No	Yes	Yes	Yes	No	Fair
Chang (2022)	Yes	Yes	Not reported	Yes	No	Yes	Yes	Yes	No	Fair
Choi (2019)	Yes	Yes	Yes	Yes	No	Yes	Yes	Yes	Yes	Good
Choi and Park (2012)	Yes	Yes	Yes	Yes	No	Yes	Yes	Yes	No	Good
Kang et al. (2007)	Yes	Yes	Not reported	Yes	No	Yes	Yes	Yes	No	Fair
Kim (2015)	Yes	Yes	Not reported	Yes	No	Yes	Yes	Yes	No	Fair
Kim et al. (2025)	Yes	Yes	Yes	Yes	No	Yes	Yes	Yes	Yes	Good
Kim and Chung (2015)	Yes	Yes	Not reported	Yes	Yes	Yes	Yes	Yes	No	Good
Kim and Yun (2009)	Yes	Yes	Yes	Yes	No	Yes	Yes	Yes	No	Good
Mun (2009)	Yes	Yes	Not reported	Yes	No	Yes	Yes	Yes	No	Fair
Mun and Koh (2009)	Yes	Yes	Not reported	Yes	No	Yes	Yes	Yes	No	Fair
So (2013)	Yes	Yes	Not reported	Yes	Yes	Yes	Yes	Yes	No	Good
Song and Jeong (2012)	Yes	Yes	Yes	Yes	No	Yes	Yes	Yes	No	Good
Yoo and Yum (2019)	Yes	Yes	Yes	Yes	No	Yes	Yes	Yes	No	Good
Yoon et al. (2014)	Yes	Yes	Yes	Yes	No	Yes	Yes	Yes	No	Good
Youn et al. (2001)	Yes	Yes	Not reported	Yes	Yes	Yes	Yes	Yes	Yes	Good
Yu (2003)	Yes	Yes	Not reported	Yes	No	Yes	Yes	Yes	No	Fair
Yu (2020)	Yes	Yes	Yes	Yes	No	Yes	Yes	Yes	No	Good

### 3.4 Pooled effect size

Table 4 presents the results of the three-level meta-analysis, comprising 512 effect sizes from 18 independent studies. The analysis yielded a statistically significant small-to-moderate pooled estimate of *r* = 0.191 (*z* = 5.127, 95% CI = [0.119, 0.261], *p* < 0.001). Heterogeneity in the estimates was observed across effect sizes (*Q*_(511)_ = 6004.06, *p* < 0.001). This variance was high (*τ^2^* = 0.039, *I^2^* = 92.46%), indicating that most of the observed variance is explained by the within-studies variance (Level 2) and/or between-studies variance (Level 3) rather than sampling error (Level 1). Following the criteria of Higgins et al. (2003), the heterogeneity test resulted in a low-to-moderate variance at Level 2 (*τ^2^* = 0.015, *I^2^* = 35.82%) and moderate variance at Level 3 (*τ^2^* = 0.024, *I^2^* = 56.64%). The statistical significance of these variance components was confirmed by the LRT, which indicated that the three-level model had a significantly better fit than the traditional two-level model (*χ^2^* = 261.06, *p* < 0.001), justifying the use of hierarchical modeling. The low-to-moderate heterogeneity at Level 2 and Level 3 suggested that the pooled effect size likely varied within and between studies, suggesting that contextual and methodological factors might influence the magnitude of the association, which called for moderator analyses.

**Table 4:** Results of the meta-analysis for the pooled effect size.

Analysis	No. studies	No. ES	*r (SE)*	95% CI	*z*	Overall			Level 2		Level 3	
						*Q(df)*	*τ^2^*	*I^2^*	L2 *τ^2^*	L2 *I^2^*	L3 *τ^2^*	L3 *I^2^*
Pooled estimate	18	512	0.191 (0.038)	[0.119, 0.261]	5.127***	6004.06 (511)***	0.039	92.46%	0.015***	35.82%	0.024***	56.64%

No., number; ES, effect sizes; CI, confidence interval; ***, *p* < 0.001.

### 3.5 Moderator and subgroup analyses

Table 5 presents the results of the moderator and subgroup analyses. The categorical moderators used in the analyses included school level, epistemic cognition construct, domain specificity, epistemic cognition measure, alignment, and learning outcome. Of the six moderators, the school level and epistemic cognition construct were found to moderate the association between epistemic cognition and learning outcomes (*p* < 0.05), while domain specificity (*F*_(1, 506)_ = 2.342, *p* = 0.126), epistemic cognition measure (*F*_(1, 510)_ = 1.984, *p* = 0.159), alignment (*F*_(1, 510)_ = 1.183, *p* = 0.277), and type of learning outcome (*F*_(3, 508)_ = 0.300, *p* = 0.828) were not. In particular, the moderator analysis showed that the school level significantly moderated the relationship between epistemic cognition and learning outcomes (*F*_(2, 485)_ = 5.683, *p* = 0.003). The subgroup analysis indicated that the elementary school had a larger effect size (*r* = 0.375, *p* < 0.001) than the middle school (*r* = 0.142, *p* = 0.001) and high school (*r* = 0.157, *p* = 0.004) groups. These findings suggest that students’ sophisticated epistemic cognition is positively associated with learning outcomes during the early stages of formal education. The moderating effects of the epistemic cognition constructs varied significantly (*F*_(7, 498)_ = 3.194, *p* = 0.002). The highest effect size was total epistemic cognition (*r* = 0.288, *p* < 0.001), followed by learning speed (*r* = 0.207, *p* < 0.001), justification (*r* = 0.187, *p* < 0.001), source (*r* = 0.174, *p* < 0.001), ability to learn (*r* = 0.174, *p* < 0.001), simplicity (*r* = 0.157, *p* = 0.001), rigid learning (*r* = 0.142, *p* = 0.011), and certainty (*r* = 0.122, *p* = 0.006).

**Table 5:** Results of moderator and subgroup analyses.

Moderator	*k*	*r*	95% CI	*z*	*F_(df1, df2)_*	*p*	L2 variance	L3 variance
School level	488				*F*_(2, 485)_ = 5.683	0.003	0.016***	0.014***
Elementary	49	0.375	[0.262, 0.477]	6.144		< 0.001		
Middle	251	0.142	[0.055, 0.226]	3.202		0.001		
High	188	0.157	[0.052, 0.259]	2.921		0.004		
EC constructs	506				*F*_(7, 498)_ = 3.193	0.002	0.015***	0.022***
Certainty	93	0.122	[0.036, 0.206]	2.77		0.006		
Simplicity	58	0.157	[0.068, 0.244]	3.444		0.001		
Source	78	0.185	[0.099, 0.268]	4.178		< 0.001		
Justification	65	0.187	[0.099, 0.271]	4.145		< 0.001		
Ability to learn	87	0.174	[0.089, 0.256]	3.983		< 0.001		
Learning speed	87	0.207	[0.123, 0.288]	4.765		< 0.001		
Rigid learning	16	0.142	[0.033, 0.247]	2.56		0.011		
Total EC	22	0.288	[0.138, 0.425]	3.691		< 0.001		
Domain specificity	508				*F*_(1, 506)_ = 2.342	0.126	0.015***	0.021***
Domain-general	81	0.281	[0.142, 0.409]	3.89		< 0.001		
Domain-specific	427	0.159	[0.081, 0.234]	3.995		< 0.001		
Alignment	512				*F*_(1, 510)_ = 1.183	0.277	0.015***	0.024***
Aligned	436	0.185	[0.111, 0.257]	4.864		< 0.001		
Non-aligned	76	0.212	[0.13, 0.291]	5.001		< 0.001		
Scale polarity	512				*F*_(1, 510)_ = 1.984	0.159	0.015***	0.023***
Bipolar	248	0.222	[0.14, 0.302]	5.2		< 0.001		
Separate	264	0.112	[−0.021, 0.241]	1.656		0.098		
Type of learning outcome	512				*F*_(3, 508)_ = 0.300	0.828	0.015***	0.024***
Higher order cognitive outcomes	184	0.188	[0.106, 0.268]	4.462		< 0.001		
Lower order cognitive outcome	55	0.208	[0.126, 0.288]	4.900		< 0.001		
Motivation/affect	109	0.189	[0.106, 0.269]	4.407		0.025		
Self-regulation	164	0.186	[0.108, 0.261]	4.647		< 0.001		

k, number of r; r, correlation coefficient; L, Level; EC, epistemic cognition; ***, *p* < 0.001.

### 3.6 Publication bias and sensitivity analysis

**Figure 2:**
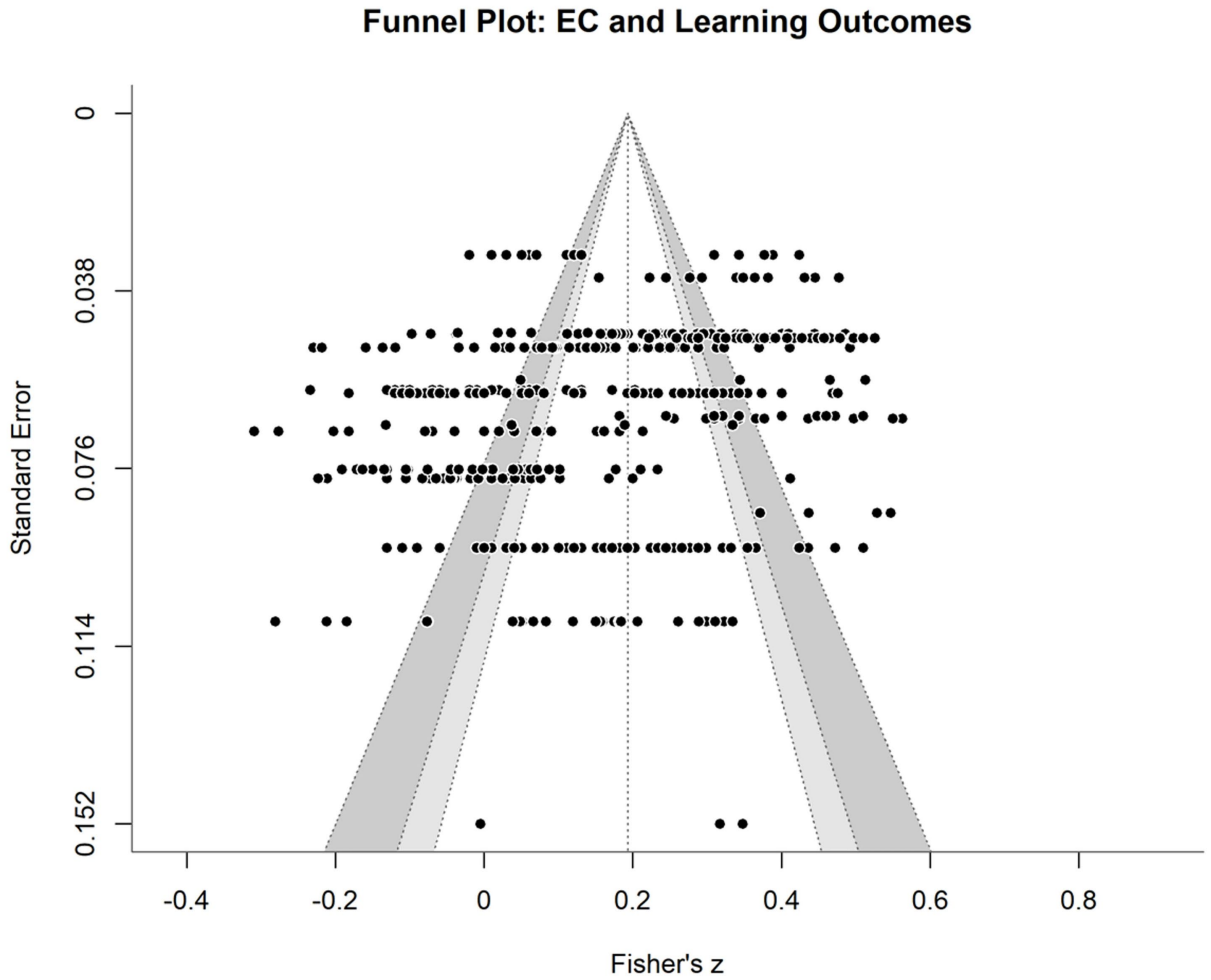
Funnel plot for effect sizes.

The funnel plot of effect sizes shown in Figure 2 appears symmetric. This visuality was supported by the results of the three-level Egger’s regression test, with a statistically insignificant slope of −1.007 (*SE* = 1.271, *z* = −0.792, *p* = 0.429). These findings imply no evidence of publication bias or a small-study effect despite the substantial heterogeneity (*τ^2^* = 0.039, *I^2^* = 92.46%). In addition, the significant intercept term of 0.257 (*SE* = 0.089, *z* = 2.896, *p* = 0.004) in the Egger’s regression test suggests that the model-implied effect size remains robust when the standard error is 0. The leave-one-out sensitivity analyses demonstrated highly stable effect sizes (*r*s = 0.177–0.203) with no influential outliers detected (|z| < 3.29).

## 4 Discussion

In this three-level meta-analysis, the pooled estimate exhibited that epistemic cognition was significantly associated with learning outcomes. In particular, two moderators, school level and epistemic cognition construct, predicted the association, while the other four, domain specificity, alignment, scale polarity, and type of learning outcome, did not. Overall, these findings suggest that sophisticated epistemic cognition should be developed and sustained from elementary through high school so that it was positively associated with multiple learning outcomes.

### 4.1 Association between epistemic cognition and learning outcomes

The small-to-moderate pooled effect size of the association between epistemic cognition and learning outcomes suggests that the more sophisticated epistemic cognition Korean students possess, the higher their learning outcomes. That is, Korean students who perceive knowledge as uncertain/tentative and complex/integrated, knowing as self-constructed and self-justified by critical assessment of knowledge sources, and learning as incremental/life-long improving and gradual appear to enhance learning outcomes. Although this finding may be limited to Korean students, this pattern is relatively consistent with the international literature. For example, Greene et al. (2018), in a meta-analysis that reviewed 132 studies conducted across countries, such as the U.S., Canada, Norway, Germany, and Türkiye, also proved that students’ epistemic cognition was associated with learning achievement at a small-to-moderate level. This consistent finding implies that this positive association is not limited to the Korean learning context. However, interpretation should be cautious, given that this study did not compare effect sizes across countries and instead focused on culturally specific epistemic cognition associated with learning outcomes (Hofer, 2016).

As the literature has argued, the positive association between epistemic cognition and learning outcomes can be fostered in a learning context in which students develop sophisticated epistemic beliefs (Muis et al., 2016). Although such a learning context was not identified due to the lack of information from the original studies, it is reasoned that the positive association was a result of interactions with a learning context. Bendixen and Rule (2004) suggested that a learning context should help students perceive cognitive disequilibrium in epistemic cognition and motivate them to change their epistemic cognition toward a more sophisticated form through various learning activities, such as reflection and social interaction. Such a learning context can be operationalized through constructivist instructional approaches (Muis et al., 2016). Previous studies have confirmed the contribution of such contexts to students’ epistemic cognition as well as learning achievement (Cartiff et al., 2021; Muis and Duffy, 2013; Rosman and Kerwer, 2022). In a meta-analysis, Cartiff et al. (2021) demonstrated that constructivist learning approaches, such as inquiry-based instruction, source evaluation, and metacognitive scaffolds, have been acknowledged as effective in improving learning achievement. These approaches involve students in the learning activities through the cultivation of sophisticated epistemic beliefs, where they transition from passive recipients to active participants by evaluating and justifying their learning processes with enhanced motivation, thereby facilitating cognitive restructuring necessary for learning achievement. Based on theoretical articulation and empirical evidence, the findings of this study suggest that sophisticated epistemic cognition should be facilitated through constructivist learning activities.

### 4.2 Results of moderator and subgroup analyses

The findings of moderator analyses demonstrated that the school level and epistemic cognition construct were key moderators of the association, whereas domain specificity, alignment, scale polarity, and type of learning outcome were not. Subsequent subgroup analyses revealed that, for school level, elementary school exhibited the highest effect size compared with middle and high schools. Elementary school seems to be a critical period when the function of epistemic cognition is more activated for learning outcomes than in middle and high schools. However, these findings were inconsistent with international research emphasizing that epistemic cognition develops with increasing age and educational level (Hofer and Pintrich, 1997; Perry, 1970). This inconsistency appears to reflect the unique education context within this culture. The learning context in Korea across different school levels often emphasizes constructivist instructional approaches, such as inquiry-based, student-centered learning, critical thinking, conceptual understanding, and problem-solving (Ministry of Education, 2022), which may create a learning context in which sophisticated epistemic cognition is more directly involved in learning. However, as students progress to higher grades, the learning context may become increasingly exam- and answer-oriented, as they generally prepare for the Sooneung, the Korean nationwide college entrance examination, in which test scores are directly linked to college admission (Youn et al., 2001). This change in the learning context appears to be accelerated by the prevalence of private tutoring. Park and Kim (2021) reported that monthly private tutoring expenditures for core school subjects, such as literature, English, mathematics, science, and social studies, were highest in high school students (approximately $240.35), followed by middle school students (approximately $199.24) and elementary school students (approximately $114.93). This assessment-oriented learning context that middle and high school students experience is likely to limit their opportunities to engage in sophisticated epistemic cognition in meaningful ways. It is possibly posited that the learning context functions as an important factor that declines the association between epistemic cognition and learning outcomes as school level increases, which should be further verified.

For the epistemic cognition construct as another significant moderator, the subgroup analyses demonstrated that all constructs, including certainty, simplicity, source, justification, ability to learn, learning speed, learning process, and total epistemic cognition, were statistically significant. The highest effect size was for total epistemic cognition, a composite score of all constructs. In fact, this finding is a direct result of the increased number of items, which enhances measurement reliability while minimizing individual observational errors, thereby yielding a correlation closer to its theoretical maximum (Spearman, 1904). The remaining epistemic cognition constructs can be grouped into the nature of knowledge (certainty, simplicity), the nature of knowing (source, justification), and the nature of knowing (ability to learn, learning speed) based on Hofer and Pintrich (1997) and Schommer (1990). For rigid learning, the beliefs can be attributed to the nature of knowledge, given that they represent an expanded view of simplicity by incorporating the knowledge acquisition process (Jehng et al., 1993).

The subgroup analyses indicated that the nature of learning had the strongest correlation, followed by the nature of knowing and the nature of knowledge, suggesting a clear hierarchy in effect sizes across these constructs. First, for the nature of learning, the ability to learn and learning speed are theoretically acknowledged as peripheral to the core epistemic cognition constructs (Hofer and Pintrich, 1997), but these beliefs may function as a proximal predictor of learning outcomes. For example, regarding beliefs about the ability to learn, Blackwell et al. (2007) in a longitudinal study revealed that students who perceived learning as incremental/life-long improved facilitated academic success, while those with beliefs about learning as entity/fixed did not, or even decreased. The nature of learning in epistemic cognition may serve as a bridge between epistemic cognition and learning outcomes.

Second, the nature of knowing (source, justification) exhibited moderate effect sizes. These constructs have been recognized as the core of epistemic cognition, reflecting how individuals use and validate knowledge and learning. The findings suggest that beliefs about sources and justification in a more sophisticated way is likely to be associated with improved learning outcomes. These beliefs measured in the included study, however, focused predominantly on authority as a source and means of justification, dichotomizing it into external authority (naïve) or internal opinion/experience (sophisticated). This reductive approach does not capture the multidimensional nature of knowing (Greene et al., 2018). In the current era of prolific online information and easy access to GenAI, in particular, a single authority is unlikely to function as a sole source of information. Rather, students are likely to rely on multiple sources of information to evaluate and justify their learning (Ferguson et al., 2013). Existing instruments that treat authority as a static, uniform entity are unlikely to capture authentic epistemic cognition regarding sources of knowledge and justification for knowing.

Lastly, the smallest effect sizes were indicated in the nature of knowledge (certainty, simplicity, rigid learning). These relatively weak associations may reflect the ontological aspects of these beliefs. Emphasizing sources of knowledge and justification for knowing as central to epistemic cognition, scholars (e.g., Chinn et al., 2011; Greene et al., 2008) have argued that epistemic cognition constructs, particularly certainty and simplicity, are more concerned with *what* knowledge is, often conceptualized in ontology, rather than *how* knowledge is acquired/learned as an epistemic stance. These constructs appear to function as a prerequisite worldview for other constructs, distal to learning outcomes (Greene et al., 2008). Consequently, this distance might yield the weakest associations between epistemic cognition constructs and learning outcomes.

Interesting findings included insignificant moderators, such as domain specificity, alignment, scale polarity, and type of learning outcome. These moderators seem not to be influential to account for the variance in the pooled effect size. Domain specificity as well as alignment have been theoretically and empirically addressed in epistemic cognition as they influence learning achievement (Greene et al., 2018; Urhahne and Kremer, 2023). One probability of these insignificant moderators is that the Korean students in the included studies might misidentify domain specificity in epistemic cognition and learning outcomes. That is, the students might have misunderstood domain-general measures as domain-specific at the moment of the data collection. For example, in Kim and Chung (2015), students were asked to complete a domain-general epistemic cognition questionnaire while responding to an instrument measuring domain-specific inquiry skills in science. This domain inconsistency between epistemic cognition and the targeted learning outcome might induce conceptual confusion among students, thereby flattening potential variance, thereby resulting in an offsetting effect of the moderators.

For scale polarity, this was a unique moderator identified in the Korean context. International studies have predominantly used bipolar, scaled instruments that force respondents to choose between a relatively sophisticated and a naïve stance (Greene et al., 2018; Urhahne and Kremer, 2023). In contrast, several Korean studies used instruments on separate scales (e.g., Bak and Chai, 2006; Choi, 2019). For example, certainty was measured through two independent items (e.g., certainty as naïve; uncertainty as sophisticated). The insignificance of this moderator suggests that measurement variance does not significantly account for heterogeneity in the pooled effect size estimate. However, using the separate scale is likely to allow for capturing students’ concurrent naïve and sophisticated epistemic beliefs, providing a novel perspective to the literature.

The statistically insignificant type of learning outcome suggests that the association between epistemic cognition and learning outcomes is relatively stable across different types of learning outcomes, including higher and lower order cognitive outcomes, self-regulation, and motivation/affect. Inconsistent with these findings, previous studies have suggested the positive association between sophisticated epistemic cognition and higher order learning achievement and motivation (Cartiff et al., 2021; Hofer, 2016). In a meta-analysis using cross-country data, Greene et al. (2018) demonstrated that students’ more availing or sophisticated epistemic cognition were significantly higher in the association with higher order learning achievement (e.g., conceptual knowledge, argumentation), compared with lower order learning achievement (e.g., declarative knowledge, procedural knowledge), although the degree of all the associations were small. This inconsistency may reflect the unique cultural characteristic in Korea, where learning is understood as a process of self-discipline and continuous effort (Choi and Kwon, 2012). Within this milieu, students’ sophisticated epistemic cognition may control their cognition and behavior, while facilitating both lower and higher order learning achievement. Consequently, sophisticated epistemic cognition may systematically align with the overarching cultural pursuit of self-discipline, thereby leading to a uniform alignment across different types of learning outcomes, such as standardized tests, conceptual change, self-regulated learning, and motivation. However, as the findings were based on correlations, not causal relationships, future research should confirm whether Korean students’ epistemic cognition consistently predicts different types of learning outcomes.

## 5 Limitations and future research

Although this study provides numerous insights into the role of epistemic cognition in learning outcomes, interpreting the findings requires caution due to five key limitations. First, the statistical power of the findings may be less stable in terms of between-variance estimates and their standard error as this study collected data from a relatively small number of studies (*n* = 18) that met the criteria. Although there is no required minimum number of studies that should be included in three-level meta-analysis, scholars have emphasized the larger number of studies to ensure the precision of variance estimates (Assink and Wibbelink, 2016; Van Den Noortgate et al., 2013). Future replication studies are suggested to incorporate a larger number of studies, encompassing diverse populations. Second, the generalizability of the findings beyond the Korean learning context is limited, as this study focused on a sample of Korean students across elementary, middle, and high schools. Although the findings suggest both culturally specific and cross-cultural aspects of epistemic cognition associated with learning outcomes among Korean students, comparative studies across cultures are necessary to determine the universal applicability of the observed patterns. Third, the findings of this study do not verify causal relationships or the structural pathways between epistemic cognition and learning outcomes. Rather, based on the findings, a hierarchy within epistemic cognition constructs and the role of motivation/affect connecting epistemic cognition with academic achievement were hypothesized. Future research should theoretically and empirically examine the hypothesized relationships with longitudinal studies, which would help clarify the mechanisms underlying these associations. Fourth, there may be unidentified moderators. While the school level and epistemic cognition construct were observed to be key moderators, the significant residual variance at Levels 2 and 3 remained stable. For example, it was expected that the learning context in which students experience would explain the variance. However, due to the lack of a description of the context, we could not empirically confirm the role of the learning context in the association between epistemic cognition and learning outcomes. Detailed descriptions of the context are required to clarify its influence, thereby helping to develop interventions that facilitate students’ sophisticated epistemic cognition. Lastly, this study does not fully account for the diverse sources of knowledge and means of justification for knowing other than authority. Future research should address these multidimensional features, both theoretically and methodologically, to capture authentic epistemic cognition.

## 6 Conclusion

Through a three-level meta-analysis, this study demonstrated a small-to-moderate effect size of the association between epistemic cognition and learning outcomes in the Korean learning context. The school level and epistemic cognition construct were found to be key moderators of the association. Overall, these findings imply that epistemic cognition is positively associated with different types of learning outcomes, such as higher and lower order cognitive outcomes, self-regulation, and motivation/affect, suggesting designing a learning context that fosters students’ sophisticated epistemic cognition. However, future research is needed to confirm whether these findings are cultural-specific or generalizable across different cultures or countries.

## Data Availability

The original contributions presented in the study are included in the article/supplementary material, further inquiries can be directed to the corresponding author.
